# Incidence of venous thromboembolism and hemorrhage related safety studies of preoperative anticoagulation therapy in hip fracture patients undergoing surgical treatment: a case–control study

**DOI:** 10.1186/s12891-016-0917-y

**Published:** 2016-02-12

**Authors:** Zhongdi Liu, Na Han, Hailin Xu, Zhongguo Fu, Dianying Zhang, Tianbing Wang, Baoguo Jiang

**Affiliations:** Department of Trauma and Orthopedics, People’s Hospital, Peking University, South Xizhimen Street No.11, Xicheng District, 100044 Beijing, China

**Keywords:** Hip fracture, Low-molecular-weight heparin, Prophylaxis, Deep vein thrombosis, Pulmonary embolism

## Abstract

**Background:**

Venous thromboembolism is a common postoperative complication following orthopedic surgeries, with morbid and potentially fatal consequences. Perioperative low-molecular-weight heparin (LMWH) therapy can reduce the incidence of venous thromboembolism, but may also increase the risk of bleeding complications. Current literature reflects the need to balance the improved efficacy of early initiating prophylaxis with increased risk of perioperative bleeding. The purpose of this study was to compare the effectiveness and hemorrhage related safety of preoperative versus postoperative LMWH therapy for prevention of deep venous thrombosis (DVT) and pulmonary embolism (PE) in hip fracture patients.

**Methods:**

We retrospectively evaluated 222 patients who underwent surgical treatment at Peking University People's Hospital between January 2009 and December 2010. Patients were allocated to two groups, receiving either preoperative or postoperative initiation of LMWH therapy for venous thromboembolism prophylaxis. Preoperative anticoagulation therapy was initiated 1–12 days prior to surgery (133 patients), and postoperative anticoagulation therapy was initiated 12 h after completion of surgery (89 patients). The preoperative group was further subdivided into four subgroups according to the time of initiation of therapy: 1–3 days before surgery (group A, 54 patients), 4–6 days before surgery (group B, 57 patients), 7–9 days before surgery (group C, 15 patients), and 10–12 days before surgery (group D, 7 patients). Occurrences of DVT, PE, adverse drug effects, intraoperative and postoperative bleeding were recorded, along with concentrations of preoperative and postoperative hemoglobin and length of hospital stay. The above parameters were compared between groups.

**Results:**

Among recipients of preoperative anticoagulation therapy, two patients developed postoperative PE (1.5 %), one patient developed DVT (0.75 %). In the group receiving postoperative initiation of anticoagulation therapy, one patient each developed PE and DVT (1.1 %, 1.1 %). There was no difference in the occurrence of PE and DVT between the two groups (*P*>0.05, Chi-square tests). We identified the incidences of major bleeding, minor bleeding, and intraspinal hematoma after spinal anesthesia, which were 0 %/0 %, 3.76 %/3.37 %, and 0 %/0 %, respectively in preoperative and postoperative anticoagulation groups. There was no significant difference in the incidence of bleeding complications between patients receiving preoperatively initiated LMWH with patients receiving postoperatively initiated LMWH. Spinal anesthesia was administered to 168 patients, with no cases of postoperative intraspinal hematoma.

**Conclusions:**

Preoperative anticoagulation therapy with LMWH may not increase intraoperative or postoperative blood loss, or the rate of intraspinal hematoma after spinal anesthesia, but also does not significantly reduce the risk of postoperative DVT or PE, compared to postoperative initiation.

## Background

Surgical intervention is the most common form of treatment for hip fractures. Surgeries like total hip arthroplasty and hip fracture fixation often lead to postoperative venous thromboembolism (VTE), associated with high mortality rates [1, 2]. For patients undergoing surgery for hip fracture, the latest guidelines for prevention of deep venous thrombosis (DVT) by the American College of Chest Physicians (ACCP) (2012, 9th edition) recommend initiation of routine subcutaneous low-molecular-weight heparin (LMWH) therapy either from at least 12 h before surgery, or at least 12 h after surgery (two to four hours after removal of the epidural catheter) [2, 3]. Perioperative LMWH therapy, although useful for preventing VTE, may increase the risk of intraoperative and postoperative bleeding [4]. The guidelines do not provide any comparisons of effectiveness and safety between preoperative and postoperative initiation of LMWH therapy. Hull et al. [5] performed a literature review and found that the timing of initiation of LMWH therapy significantly affected the effectiveness of DVT prophylaxis. In another review, Perka [6] reported no difference in the effectiveness of preoperative versus postoperative initiation of anticoagulation therapy for DVT prophylaxis. It is important to take into consideration both effectiveness and safety of the regimes when determining the optimal time for initiation of anticoagulation therapy in patients undergoing orthopedic surgeries for which DVT prophylaxis is indicated [7, 8]. Preoperative initiation of anticoagulation therapy can effectively reduce the risk of DVT, but, on the other hand, may increase the risk of intraoperative and postoperative bleeding [9, 10]. Further, among patients receiving spinal anesthesia, preoperative initiation of anticoagulation therapy may increase the risk of intraspinal hematoma. Postoperative initiation of anticoagulation therapy reduces the likelihood of bleeding complications, but may not be as effective as preoperative regimen in preventing DVT. Thus, it is not surprising that consensus on optimal time for initiation of anticoagulation therapy is yet to be reached [11, 12]. Regardless of the drug used, benefits (prevention of VTE) and risks (bleeding and intraspinal hematoma) should be taken into account while deciding whether to administer anticoagulation therapy [13]. Such decision making depends on many parameters such as type of drug, drug dosage, timing of drug administration, surgical procedure, medical history, condition of the patient etc. In clinical practice, the timing of initiation of anticoagulation therapy varies widely across geographical region [5, 14]. This retrospective study analyzed outcomes of perioperative anticoagulation therapy in 222 patients who underwent surgery for treatment of femoral neck or intertrochanteric fractures, between January 2009 and December 2010 at Peking University People’s hospital, to evaluate the effectiveness and safety of pre- and postoperative initiation of anticoagulation therapy for DVT prophylaxis. As the optimal regimen is uncertain because direct comparisons among different regimens with sufficient large sample sizes are not available, our study may provide a reference in choosing the appropriate methods to prevent the perioperative venous thromboembolism.

## Methods

### Patients

This study enrolled hip fracture patients who underwent surgical treatment between January 2009 and December 2010 in the Department of Orthopedics and Trauma at Peking University People's Hospital. Study subject selection criteria were determined so as to avoid the influence of known risk factors on outcomes [15, 16]. The inclusion criteria were: (1) surgical treatment for hip fracture, especially total hip arthroplasty; (2) age >40 years; (3) weight 40–100 kg; (4) no preoperative signs/symptoms of DVT such as lower limb swelling, pain, stiffness, superficial venous congestion, or increased skin temperature; (5) no preoperative signs/symptoms of pulmonary embolism (PE) such as dyspnea, chest pain, or cough; and, (6) VTE prophylaxis with LMWH. Patients with multiple fractures, hemorrhagic diseases or other contraindications to anticoagulation therapy (e.g., those taking oral anticoagulants, or platelet inhibitors, experiencing any active bleeding; having history of heparin-induced thrombocytopenia, platelet count <100,000/mm^3^etc.) were excluded. As per the ACCP guidelines, all included patients were in the high-risk group for VTE [1]. Occurrences of deep venous thrombosis, pulmonary embolism, adverse drug effects, intraoperative and postoperative bleeding were recorded, along with concentrations of preoperative and postoperative hemoglobin, blood loss, post-surgical wound drainage, duration of surgery and length of hospital stay. The above parameters were compared between groups.

### Ethics statement

Written informed consent was obtained from all participants, and the study was approved by the Ethics Committee of People's Hospital, Peking University. All clinical investigations are conducted according to the principles expressed in the Declaration of Helsinki.

### Prevention of VTE

Study subjects were categorized into two groups according to time of initiation of LMWH therapy --- 1) preoperative and 2) postoperative. Patients in both groups received subcutaneous LMWH (Fraxiparine; GlaxoSmithKline, Notre Dame de Bondeville, France), 4100 units daily until discharge. The timing of initiation of therapy was based on respective physician’s preference, and not on the preoperative condition of the patient. Preoperative anticoagulation therapy was initiated 1–12 days prior to surgery, and postoperative anticoagulation therapy was initiated 12 h after completion of surgery. The preoperative group was further subdivided into four subgroups according to the time of initiation of therapy: 1–3 days before surgery (group A), 4–6 days before surgery (group B), 7–9 days before surgery (group C), and 10–12 days before surgery (group D). All study patients were subjected to basic preventive measures, including care to avoid of intraoperative injury to the intima, regular postoperative turning, early functional exercises, and elastic stockings. Drains were removed when the drainage volume got reduced to <50 mL/24 h. The mean duration of hospitalization was 20±5 days in patients who received preoperative anticoagulation therapy and 19 ± 6 days in patients receiving only postoperative anticoagulation therapy. Patients were encouraged to walk following discharge in order to reduce the risk of DVT.

### Method of anesthesia

The American Academy of Orthopedic Surgeons (AAOS) guidelines recommend spinal, epidural, or combined spinal-epidural anesthesia for patients undergoing total hip or total knee arthroplasty [17]. Preoperative antithrombotic and antiplatelet therapy is often administered to orthopedic patients to prevent postoperative DVT [18]. Although such prophylactic measures have been reported to increase the risk of intraspinal hematoma, it has also been shown that with appropriate dosage and timing, anticoagulation therapy can prove to be safe and effective in patients undergoing spinal anesthesia [19]. In our study, 168 patients received spinal anesthesia; while 54 patients did not – owing to structural deformity of vertebral column or previous thoracolumbar trauma. Preoperative anticoagulation therapy was not considered as a contraindication to spinal anesthesia.

### Effectiveness of LMWH therapy

Patients in both groups underwent routine preoperative and postoperative blood tests including liver and kidney function tests, and urine tests. Specific investigations for DVT were conducted if any of the patients developed sudden onset of lower limb swelling, pain, numbness, superficial venous congestion, or increase in skin temperature. Investigations for PE were undertaken if anyone developed dyspnea, chest pain, hemoptysis, or cyanosis. D-dimer levels were also assessed for patients with suspected VTE. Lower limb compression color Doppler ultrasonography and computed tomography pulmonary angiography were performed as needed. Positive imaging findings were mandatory requirements for definitive diagnosis of DVT.

### Safety of LMWH therapy

Medical records of study patients including postoperative wound drainage, drain removal time, volumes of wound bleeding, hematoma formation, and changes in lower limb sensation or muscle strength were reviewed. Preoperative (the day before surgery) and postoperative (the day after surgery) laboratory test results were compared to detect presence of major bleeding, thrombocytopenia, and any significant changes in organ function. Major bleeding was defined as any of the following: decrease in hemoglobin concentration by 20 g/L or more within 24 h, need for transfusion of more than 2 units of red blood cells based on change in hemoglobin concentration, evidence of major organ bleeding such as intracranial or gastrointestinal bleeding, intraoperative bleeding needing surgical intervention, or intramuscular bleeding resulting in compartment syndrome [20]. All other types of bleeding were classified as minor. Thrombocytopenia was defined as either a reduction in platelet concentration from >150 × 10^19^/L (preoperative) to <100 × 10^19^/L (postoperative), or a >50 % decrease during postoperative period from a preoperative concentration of <150 × 10^19^/L [21]. Transaminase levels and signs of allergic reactions were also recorded for every study patient.

### Statistical analysis

Pre- and postoperative anticoagulation therapy groups, and subgroups among the recipients of preoperative anticoagulation therapy, were compared using the Chi-square tests and Non-parametric tests. Analyses were performed using Statistical Product and Service Solutions statistical software (Spss19.0). A value of *P* < 0.05 was considered statistically significant.

## Results

### Patient characteristics

A total of 222 patients (81 males, 141 females) met the criteria for inclusion, of whom 111 were admitted with femoral neck fractures and 111 with intertrochanteric fractures. The mean age of patients was 75 ± 8 (S.D.) years. Among study patients, 81, 30 and 111 underwent total hip arthroplasty, hemi-arthroplasty, and fixation with proximal femoral nail anti-rotation, respectively. 23 patients received combined spinal-epidural anesthesia, while 145 and 54, respectively, were subjected to spinal and general anesthesia. Table 1 shows the group characteristics among recipients of pre- and postoperative anticoagulation therapy. Table 2 depicts patient characteristics in the subgroups with received initiation of anticoagulation therapy 1–3 days before surgery (group A), 4–6 days before surgery (group B), 7–9 days before surgery (group C), and 10–12 days before surgery (group D).

**Table 1:** Characteristics of patients who received preoperative and postoperative initiation of anticoagulation therapy

Characteristics	Preoperative anticoagulation group	Postoperative anticoagulation group	Total	*P* value
	(*n*=133)	(*n*=89)	(n=222)	
Basic Information				
Age(years), mean ± SD	76±9	73±14	75±8	0.396
Gender				
Male	52	29	81	
Female	81	60	141	
Body weight (kg), mean ± SD	58.87±11.34	58.76±10.57	58.83±11.01	
Body mass index, mean ± SD	22.20±3.58	22.32±3.80	22.25±3.50	0.506
Comorbid conditions, n (%)				
Hypertension	36 (27.06)	20 (22.47)	56 (25.23)	
Diabetes	33 (24.81)	17 (19.10)	50 (22.53)	
Congestive heart failure	5 (3.76)	1 (1.12)	6 (2.70)	
Varicose veins	4 (3.01)	4 (4.49)	8 (3.60)	
Currently active cancer	0 (0)	0 (0)	0 (0)	
Paralysis of lower limbs	7 (5.26)	3 (3.37)	10 (4.5)	
Atrial fibrillation	11 (8.27)	4 (4.49)	15 (6.76)	
Prior stroke or TIA	7 (5.26)	5 (5.62)	12 (5.41)

**Table 2:** Patients’ characteristics according to the time of initiation of preoperative anticoagulation therapy

Characteristics	Group A	Group B	Group C	Group D	Total	*P* value
	(*n*=54)	(*n*=57)	(*n*=15)	(*n*=7)	(*n*=133)	
Basic Information						
Age (years),mean ± SD	76±11	75±8	76±8	79±4	76±9	0.647
Gender						
Male	22	20	7	3	52	
Female	32	37	8	4	81	
Body weight (kg), mean ± SD	58.31±11.55	58.96±12.01	61±9.96	57.71±9.36	58.87±11.34	
Body mass index, mean ± SD	22.08±3.94	22.28±3.68	22.46±2.71	22.08±3.94	21.83±58.10	0.971
Comorbid conditions, n (%)						
Hypertension	11 (20.37)	13 (22.81)	8 (53.33)	4 (57.14)	36 (27.06)	
Diabetes	9 (16.67)	14 (24.56)	7 (46.67)	5 (71.43)	33 (24.81)	
Congestive heart failure	3 (5.56)	1 (1.75)	0 (0)	1 (14.29)	5 (3.76)	
Varicose veins	2 (3.70)	1 (1.75)	1 (6.67)	0 (0)	4 (3.01)	
Currently active cancer	0 (0)	0 (0)	0 (0)	0 (0)	0 (0)	
Paralysis of lower limbs	2 (3.70)	4 (7.02)	1 (6.67)	0 (0)	7 (5.26)	
Atrial fibrillation	5 (9.26)	4 (7.02)	1 (6.67)	1 (14.29)	11 (8.27)	
Prior stroke or TIA	2 (3.70)	4 (7.02)	1 (6.67)	0 (0)	7 (5.26)	

### Treatment data

The mean duration of surgery was 176 ± 34 min. Mean blood loss was estimated to be 216 ± 135 mL, with 15 patients requiring intraoperative blood transfusion and 93 needing postoperative blood transfusion. Mean post-surgical wound drainage, collected from tubes placed according to surgical conventions, amounted to 225 ± 133 mL and mean time till drain removal was 2 days, while mean length of hospital stay was 20 ± 4 days.

### Incidence of complications

Among recipients of preoperative anticoagulation therapy, two patients developed postoperative PE, of whom one died and one was successfully treated by thrombolysis. Only one patient developed DVT in the preoperative group, and he was successfully treated with thrombolysis and supportive measures. In the group receiving postoperative initiation of anticoagulation therapy, one patient each developed PE and DVT, both of whom recovered with symptomatic treatment only. There was no difference in the occurrence of PE and DVT between the two groups (*P*>0.05, Chi-square tests).

In the preoperative group, five patients suffered from minor wound bleeding, indicated by wound ecchymosis and bleeding. In the same group, one patient developed a suspected allergic skin reaction, characterized by rashes over abdomen and lower limbs, but no change in heart rate or blood pressure. The condition was treated successfully with medication. In the group receiving postoperative anticoagulation therapy only, three patients developed minor wound bleeding, one suffered from suspected heparin-induced thrombocytopenia (successfully treated by platelet transfusion and symptomatic treatment), and one had a mild increase in serum transaminase levels. None of the study patients required discontinuation of LMWH therapy. Spinal anesthesia was administered to 168 patients, with no cases of postoperative intraspinal hematoma (Table 3, Table 4).

**Table 3:** Surgical and postoperative information, efficacy and safety in patients receiving preoperative and postoperative initiation of anticoagulation therapy

Characteristics	Preoperative Anticoagulation group	Postoperative anticoagulation group	Total	*P* value
	(*n*=133)	(*n*=89)	(*n*=222)	
Surgical information				
General anaesthesia, n (%)	37 (27.82)	17 (19.10)	54 (34.32)	
Surgery time (min),mean ± SD	173±43	180±53	176±34	0.287
Intraoperative bleeding (mL),mean ± SD	208±183	228±179	216±135	0.204
Intraoperative blood transfusion, n (%)	9 (6.77)	6 (6.74)	15 (6.76)	
Postoperative information				
Postoperative blood transfusion, n (%)	22 (16.54)	13 (14.61)	35 (15.77)	
Postoperative drainage (mL),mean ± SD	212±154	244±196	225±133	0.403
Reduction in hemoglobin concentrations (g/L)	23±11	27±13	25±14	0.143
Hospital stays(days),mean ± SD	20±5	19±6	20±4	0.104
Efficacy assessment, n (%)				
Symptomatic DVT	1 (0.75)	1 (1.1)	2 (0.90)	
Symptomatic PE	2 (1.5)	1 (1.1)	3 (1.35)	
Safety assessment, n (%)				
Major bleeding	0 (0)	0 (0)	0 (0)	
Minor bleeding	5 (3.76)	3 (3.37)	8 (3.60)	
Thrombocytopenia	0 (0)	1 (1.1)	1 (0.45)	
Elevated aminotransferase	0 (0)	1 (1.1)	1 (0.45)	
Suspicious allergic reaction	1 (0.75)	0 (0)	1 (0.45)

**Table 4:** Surgical and postoperative information, efficacy and safety in each subgroup of patients receiving preoperative initiation anticoagulation group

Characteristics	Group A	Group B	Group C	Group D	Total	*P* value
	(*n*=54)	(*n*=57)	(*n*=15)	(*n*=7)	(*n*=133)	
Surgical information						
General anaesthesia, n (%)	14 (25.93)	16 (28.07)	5 (33.33)	2 (28.57)	37 (27.82)	
Surgery time (min),mean ± SD	171±51	175±38	168±37	187±21	173±43	0.408
Intraoperative bleeding (mL),mean ± SD	210±89	211±171	227±234	192±142	208±183	0.805
Intraoperative blood transfusion, n (%)	4 (7.41)	2 (3.51)	2 (13.33)	1 (14.29)	9 (6.77)	
Postoperative information						
Postoperative blood transfusion, n (%)	6 (11.11)	9 (15.79)	4 (26.67)	3 (42.86)	22 (16.54)	
Postoperative drainage (mL),mean ± SD	201±157	203±149	259±188	260±81	212±154	0.277
Reduction in hemoglobin concentrations (g/L)	24±11	24±10	18.±14	15±10	23±11	0.375
Hospital stays(days),mean ± SD	18±4	21±4	24±7	23±7	20±5	0.00
Efficacy assessment, n (%)						
Symptomatic DVT	0 (0)	1 (1.75)	0 (0)	0 (0)	1 (0.75)	
Symptomatic PE	1 (1.85)	0 (0)	1 (6.67)	0 (0)	2 (1.50)	
Safety assessment, n (%)						
Major bleeding	0 (0)	0 (0)	0 (0)	0 (0)	0 (0)	
Minor bleeding	1 (1.85)	0 (0)	2 (13.3)	2 (28.6)	5 (3.76)	
Thrombocytopenia	0 (0)	0 (0)	0 (0)	0 (0)	0 (0)	
Elevated aminotransferase	0 (0)	0 (0)	0 (0)	0 (0)	0 (0)	
Suspicious allergic reaction	0 (0)	1 (1.75)	0 (0)	0 (0)	1 (0.76)	

## Discussion

Patients undergoing hip fracture surgery are at increased risk of VTE, especially in those with additional risk factors. Without prophylaxis, rate of postoperative VTE can be as high as 40–60 %, with no major differences in rates between Asian and Western countries [1, 22]. Chemical prophylaxis in these patients was considered appropriate. In spite of that, bleeding complications was the major concern of surgeons during chemoprophylaxis, and should be considered when deciding on the time of initiation of therapy [23, 24]. A literature review by Dahl et al. [4] found that, among patients undergoing total hip arthroplasty, the dose, administration time (preoperative initiation of 40 mg daily vs. postoperative initiation of 30 mg twice daily) and duration of use of enoxaparin (5–14 days vs. 28–39 days) were not associated with major bleeding events. In the present study, there was no occurrence of major hemorrhagic complications, and only eight cases of wound bruising or oozing, suggesting that administration of LMWH is quite safe. Previous reports have indicated that adverse reactions of LMWH are low, and no serious adverse reactions were observed in this study. One case had a mild elevated aminotransferase level and one had a suspicious allergic reaction, but LMWH was not discontinued in any of these cases. For the patient with a suspicious allergic reaction, no further examinations were taken since the skin rash disappeared after topical drug treatment.

This study included patients at high risk of VTE, who received regular LMWH administration along with adjunct mechanical therapy measures. Preoperative anticoagulation therapy was initiated 1–12 days prior to surgery, as soon as the patient was admitted to hospital, and postoperative anticoagulation therapy was initiated 12 h after completion of surgery. Drain removal was 3 h prior to LMWH administration, thus reducing the risk of bleeding during the removal process. DVT and PE diagnosis during anticoagulation treatment were made strictly in accordance with the patient's clinical manifestations and laboratory test results, with suspicious cases undergoing lower limb compression color Doppler ultrasonography or computed tomography pulmonary angiography.

Both ACCP [“Prevention of Venous Thromboembolism in Orthopedic Surgery Patients: Antithrombotic Therapy and Prevention of Thrombosis” (9th edition, 2012)] and AAOS guidelines [“Clinical Practice Guideline on Preventing Venous Thromboembolic Disease in Patients Undergoing Elective Hip and Knee Arthroplasty” (2011)] recommend anticoagulation therapy for DVT prophylaxis for patients undergoing hip or knee arthroplasty or surgical treatment of hip fractures [2, 17]. The ACCP and AAOS guidelines also suggest LMWH as the preferred agent for DVT prophylaxis [1]. It can be noted that LMWH is the most commonly used drug for the prevention of postoperative DVT in the United States [25]. The most common time of initiation of anticoagulation therapy was reported to be 13–24 h after surgery (35 %). Mont M [26] highlighted that preoperative initiation of anticoagulation therapy had been based on a theory that ascribe surgical trauma as the principal cause of DVT, and postoperative initiation of prophylaxis might be adequate because DVT usually develops over a prolonged period of time (several days to weeks). They further argued that postoperative initiation of prophylaxis should be favored as it reduced the risks associated with spinal anesthesia and surgery.

Some researchers have recommended against preoperative initiation of anticoagulation therapy under the assumption that it may increase the rate of postoperative intraspinal hematoma in patients receiving spinal anesthesia. Occurrence of spinal cord edema following spinal anesthesia is quite rare (1:150 000), and the risk of spinal hematoma in women undergoing knee arthroplasty is only 1:3600 [23, 27]. Hull et al. performed a meta-analysis of studies on effectiveness and safety of preoperative and postoperative initiation of LMWH therapy in elective hip joint replacement surgery patients. The study found that, among patients undergoing preoperative and postoperative initiation of therapy, the incidence of DVT were 10 % and 15.3 %, respectively (*P* = 0.02), and, contrary to expectation, the incidence of major bleeding events was lower in patients with preoperative rather than postoperative initiation of therapy (0.9 % vs 3.5 %, *P* = 0.01) [24].

Our study did not detect any significant differences in the effectiveness of DVT or PE prevention between initiation of LMWH therapy ≥12 h before surgery and ≥12 h after surgery. No cases of postoperative intraspinal hematoma were detected among the 168 study patients who received spinal anesthesia for total hip arthroplasty or hip fracture fixation surgery. Preoperative initiation of anticoagulation therapy did not increase the risk of intraspinal hematoma. Moreover, no significant differences were detected in terms of intraoperative blood loss, postoperative wound drainage, change in hemoglobin concentration after surgery, and change in hematocrit after surgery among subgroups of patients in whom anticoagulation therapy were initiated 1–3 days, 4–6 days, 7–9 days, and 10–12 days before surgery.

Our study has several limitations. First, this is a single-centered, retrospective observational study subject to inherent biases including unmeasured confounding, selection, and ascertainment bias. Second, the patients enrolled in the study have different fracture types, femoral neck fractures (Garden III-IV), intertrochanteric fractures (Evans-JensenI-V); it is possible that complex fractures may affect the final result, associated with an increase in operation time and a higher postoperative morbidity. Third, we identified potential DVT cases on the basis of clinical features, e.g. lower extremity swelling, pain, stiffness, superficial venous congestion, and increase in skin temperature, without performing routine angiography or color Doppler ultrasonography in all patients. This may have resulted in failure to detect DVT cases with minimal or no clinical signs [28], thereby reducing the validity of outcome data. Fourth, the sample size of the groups and subgroups is not great. Finally, post-discharge medical record follow-up was imperfect, therefore, it is possible that some venous thromboembolism events may have occurred after hospital discharge and were missed.

## Conclusions

Although early initiation of anticoagulation therapy is always regarded as more effective in reducing risk of VTE, and postoperative initiation of anticoagulation therapy is considered safer than preoperative initiation, the above results indicate that preoperative initiation of anticoagulation therapy with LMWH may not increase intraoperative or postoperative risk of bleeding, but also does not significantly reduce the risk of postoperative DVT or PE, compared to postoperative initiation. Our study may provide a reference in choosing the appropriate methods to prevent the perioperative venous thromboembolism, but further studies with more accurate diagnostic methods may pave the way for evidence-based intervention in this regard.

## Abbreviations

LMWH: Low-molecular-weight heparin
DVT: Deep venous thrombosis
PE: Pulmonary embolism
VTE: Venous thromboembolism
ACCP: American College of Chest Physicians
